# Nano MiRNA‐Functionating Tetrahedral Framework Nucleic Acid for Cartilage‐Targeted Ferritinophagy Modulation to Attenuate Temporomandibular Joint Osteoarthritis

**DOI:** 10.1002/smsc.202500267

**Published:** 2025-12-12

**Authors:** Wenxiu Yuan, Maotuan Huang, Linxin Chen, Sihang Chen, Hanyu Lin, Nengwen Huang, Yifeng Xing, Chengchaozi Wang, Jie Lu, Min Fu, Linyu Xu, Jiang Chen

**Affiliations:** ^1^ Postdoctoral workstation and Fujian Key Laboratory of Oral Diseases & Fujian Provincial Engineering Research Center of Oral Biomaterial & Stomatological Key Lab of Fujian College and University School and Hospital of Stomatology Fujian Medical University Fuzhou 350002 China; ^2^ Department of Orthodontics School and Hospital of Stomatology Fujian Medical University Fuzhou 350002 China; ^3^ Department of Hepatobiliary Surgery and Fujian Institute of Hepatobiliary Surgery Fujian Medical University Union Hospital Fujian Medical University Fuzhou 350000 China; ^4^ Institute of Stomatology and Research Center of Dental and Craniofacial Implants School and Hospital of Stomatology Fujian Medical University Fuzhou 350002 China

**Keywords:** ferritinophagy, microRNA delivery, MiR‐143‐3p, temporomandibular joint osteoarthritis, tetrahedral framework nucleic acids

## Abstract

Current clinical interventions lack effective strategies to arrest temporomandibular joint osteoarthritis (TMJOA) progression. Emerging evidence highlights the therapeutic potential of microRNAs (miRNAs) in osteoarthritis (OA) management, though critical challenges persist regarding delivery efficiency, including unsatisfactory cellular uptake, immunogenicity, and structural instability of miRNA‐based therapeutics. Considering the powerful editability of tetrahedral framework nucleic acids (tFNAs) for gene delivery, a novel nanoscale gene delivery system using tetrahedral framework nucleic acids functionalized with miR‐143‐3p (tFNAs‐143) is engineered. Through comprehensive in vivo modeling of TMJOA destabilization and in vitro simulation of IL‐1β‐induced inflammatory microenvironments, the therapeutic efficacy and molecular mechanisms underlying OA pathophysiology of tFNAs‐143 are systematically investigated. The results demonstrate that tFNAs‐143 exhibits excellent cellular internalization in chondrocytes and effectively mitigates TMJOA progression by impeding ferritinophagy‐mediated ferroptosis. This study advances miRNA delivery technology for TMJOA therapy, deepens the insights into TMJOA pathogenesis, and proposes a promising nanotherapeutic strategy for developing targeted TMJOA therapies.

## Graphical abstract

Current temporomandibular joint osteoarthritis (TMJOA) treatments lack progression control. MicroRNAs (miRNAs) offer therapeutic potential but face delivery hurdles. A novel nanodelivery system is developed using tetrahedral framework nucleic acids functionalized with miR‐143‐3p (tFNAs‐143). Tested in TMJOA models and inflammatory conditions, tFNAs‐143 mitigate TMJOA progression by inhibiting ferritinophagy‐mediated ferroptosis. This advances both TMJOA treatment and pathological understanding.

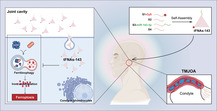

## 1 Introduction

Temporomandibular disorders (TMDs), a heterogeneous group of musculoskeletal conditions affecting the temporomandibular joint (TMJ) and associated neuromuscular structures, afflict over 15% of adults globally and constitute a predominant etiology of chronic orofacial pain.^[^ ^1^ ^]^ Among TMD subtypes, osteoarthritis (OA)‐like degenerative changes in the TMJ,^[^ ^2^ ^]^ termed temporomandibular joint osteoarthritis (TMJOA), are characterized by cartilage degradation, aberrant subchondral bone remodeling, and synovial inflammation.^[^ ^3^ ^]^ The condylar cartilage of the TMJ is different from other joint cartilage. It is a fibrocartilage composed of collagen type I and collagen type II, and it is difficult to restore normal tissue structure after injury. Progressive TMJOA leads to mandibular dysfunction, impaired mastication, and debilitating pain, resulting in substantial physical/psychological morbidity and socio‐economic burdens.^[^ ^4^ ^]^ Given the current lack of clarity regarding TMJOA etiology and mechanisms, so TMJOA management predominantly focuses on symptomatic treatment,^[^ ^4^, ^5^, ^6^ ^]^ relying on analgesics, intra‐articular injections, and surgery. Drug therapy involves anti‐inflammatory drugs,^[^ ^7^ ^]^ joint lubricants,^[^ ^8^ ^]^ and painkillers,^[^ ^9^ ^]^ but these drugs cannot effectively block the progression of TMJOA, and long‐term use has significant side effects. In addition, only a very small number of patients with severe symptoms are treated with expensive surgical procedures such as joint replacement.^[^ ^3^, ^10^ ^]^ These conventional approaches neither arrest disease progression nor avoid potential iatrogenic complications. Consequently, identifying disease‐modifying strategies targeting TMJOA pathogenesis remains an urgent clinical priority.

The Nobel Prize in Physiology or Medicine 2024 highlighted the groundbreaking role of microRNAs (miRNAs) in gene regulation,^[^ ^11^, ^12^ ^]^ reinvigorating interest in their therapeutic potential. Prior investigations employing high‐throughput RNA sequencing identified 142 differentially expressed miRNAs on cartilage preservation areas and cartilage injury areas, underscoring their central involvement in OA pathophysiology.^[^ ^13^ ^]^ Notably, reduced miR‐143‐3p expression in rheumatoid arthritis correlates with chronic inflammatory.^[^ ^14^ ^]^ Emerging evidence indicates that miR‐143‐3p regulates synovial cell proliferation and apoptosis,^[^ ^15^ ^]^ and miR‐143‐3p agonist ameliorates OA‐associated pathology.^[^ ^16^ ^]^ Nevertheless, the underlying mechanisms of miR‐143‐3p in TMJOA progression remains incompletely characterized. Moreover, transport challenges persist in miRNA therapeutics, particularly regarding safe/efficient delivery to target sites. At present, miRNA delivery systems mainly include applications of synthetic mimics, lipid nanoparticles, virus vector delivery, etc.^[^ ^17^, ^18^, ^19^ ^]^ However, current strategies have drawbacks that cannot be ignored, such as poor membrane penetration of miRNA mimics leading to low cell absorption rates, easy clearance, and frequent application.^[^ ^17^ ^]^ The high immunogenicity of liposomes often activates the host immune response.^[^ ^20^ ^]^ Viral vectors have significant safety issues and nonspecific adverse reactions.^[^ ^21^ ^]^ The exploration of novel gene delivery systems and nano‐therapy systems has aroused the interest of many researchers and provided a new direction for miRNA delivery.^[^ ^22^, ^23^ ^]^ Tetrahedral framework nucleic acids (tFNAs), rigid 3D nanostructures with exceptional biocompatibility, membrane permeability, and programmable cargo‐loading capacity, offer a transformative solution for miRNA delivery.^[^ ^24^, ^25^ ^]^ TFNA‐loaded evodiamine inhibits chondrocyte apoptosis,^[^ ^26^ ^]^ while miR‐214‐tFNAs trigger tumor cell apoptosis via *Survivin* downregulation.^[^ ^27^ ^]^ Based on these findings, is it possible to use tFNA to load miR‐143‐3p and construct miR‐143‐3p functionalized tFNA (tFNAs‐143) to improve delivery efficiency while minimizing immunogenicity. This nanoplatform may potentially overcome current limitations by leveraging tFNAs’ structural stability and cargo‐protection capabilities.

TMJOA pathogenesis initiates with extracellular matrix (ECM) degradation in cartilage, driven by catabolic processes.^[^ ^28^ ^]^ Recent advances reveal that D‐mannose mitigates OA through HIF‐2α‐mediated ferroptosis inhibition and ECM metabolism regulation.^[^ ^29^ ^]^ Ferroptosis is a regulated cell death pathway marked by glutathione depletion, lipid peroxidation, and iron dysregulation.^[^ ^30^ ^]^ Ferritinophagy, a selective autophagy process mediated by nuclear receptor coactivator 4 (NCOA4), governs intracellular iron homeostasis and serves as an upstream trigger for ferroptosis.^[^ ^31^, ^32^ ^]^ Notably, Li found that lncRNA SLC16A1‐AS1/miR‐143‐3p/SLC7A11 axis mediated the ferroptosis of renal cell carcinoma.^[^ ^33^ ^]^ Intriguingly, miR‐143‐3p agonist reversed TMJOA‐induced GPX4 suppression.^[^ ^16^ ^]^ SEMA5A‐IT1‐packaged small extracellular vesicles upregulated SLC7A11/BCL2 through miR‐143‐3p sponging and reduced ferroptosis.^[^ ^34^ ^]^ Contrastingly, it is worth noting that exosomal miR‐143‐3p promoted autophagy via CHK2‐Beclin2 pathway.^[^ ^35^ ^]^ Furthermore, miR‐143‐3p suppressed ectopic stromal cell proliferation by downregulating autophagic activity.^[^ ^36^ ^]^ These dual regulatory roles position miR‐143‐3p at the nexus of ferroptosis‐autophagy crosstalk, yet whether the miR‐143‐3p functionalized nucleic acid tadpoles (tFNAs‐143) can regulate ferritinophagy and inhibit ferroptosis to alleviate TMJOA and its role in TMJOA‐associated ECM dysregulation remains undefined.

In this study, we developed a nano‐miR‐143‐3p functionalized tetrahedral framework nucleic acids (tFNAs‐143) system and established destabilization of unilateral anterior crossbite (UAC)‐induced TMJOA model as well as in vitro TMJOA simulation system induced by IL‐1β exposure, to investigate the effect of tFNAs‐143 in TMJOA, together with molecular mechanisms underlying OA pathophysiology. Additionally, high‐throughput RNA sequencing and bioinformatics techniques were used to delineate tFNAs‐143's mode of action in TMJOA treatment. We anticipate our findings will advance miRNA delivery technology for TMJOA treatment, deepen the theoretical understanding of the pathogenesis of TMJOA, and provide a theoretical foundation for developing novel therapeutic strategies for TMJOA.

## 2 Results

### 2.1 Construction and Characterizations of TFNAs and TFNAs‐143

**Figure 1:**
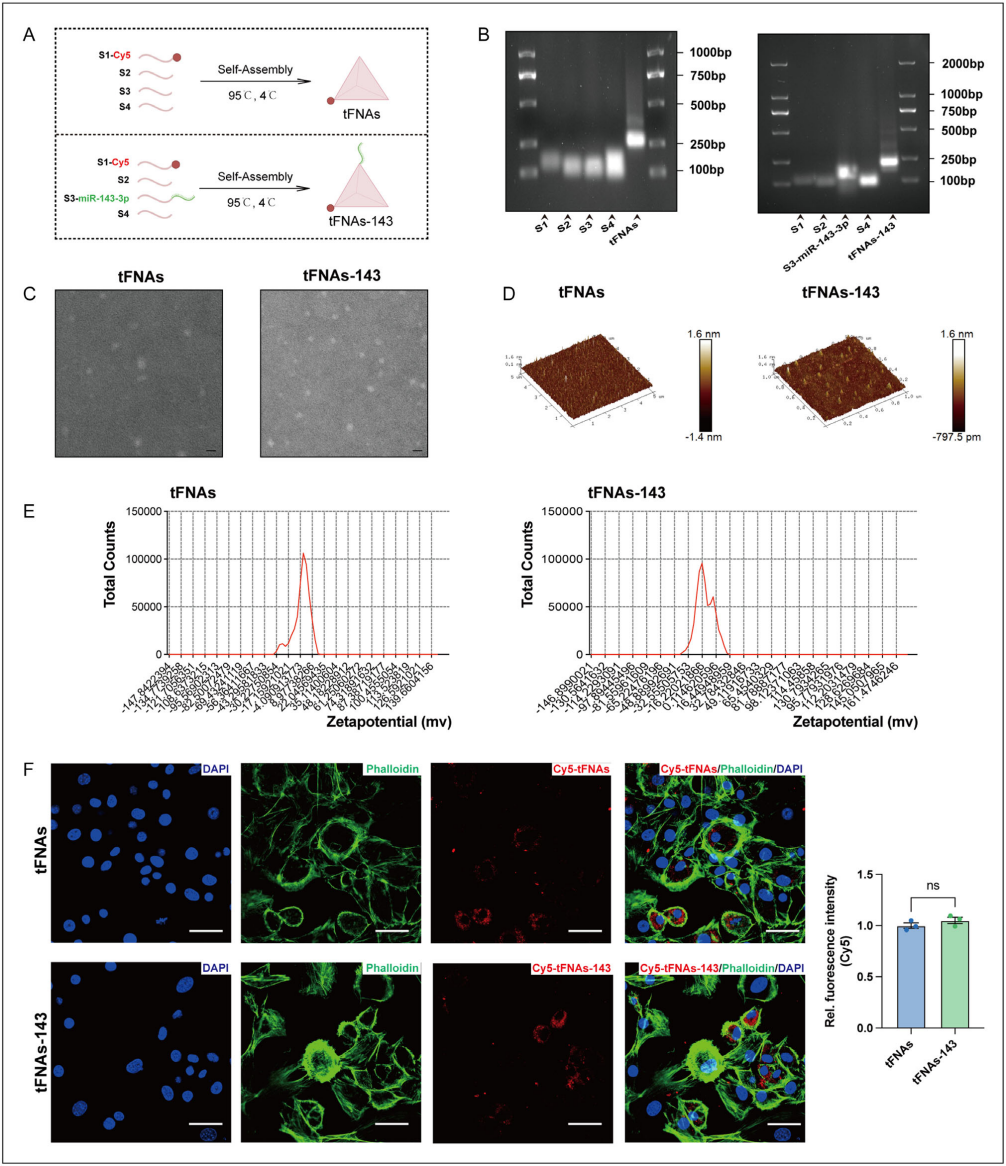
Construction and characterizations of tFNAs and tFNAs‐143. A) Schematic diagram of tFNAs and tFNAs‐143 synthesis. B) Polypropylene acyl amine gel electrophoresis demonstrating the successful production of tFNAs and tFNAs‐143. C) TEM images of tFNAs and tFNAs‐143. Scale bar: 100 nm. D) AFM images of tFNAs and tFNAs‐143. Scale bar: 400 nm. E) Zeta potential test of tFNAs and tFNAs‐143. F) Immunofluorescence showing the uptake of tFNAs or tFNAs‐143 by chondrocytes. Two‐tailed Student's *t* tests for comparison between two groups. Scale bar: 25 μm, ns, not significant.

As shown in **Figure** **1**A, tFNAs‐143 consisted of four isometric single‐strand DNAs (ssDNA), with miR‐143‐3p modified as the vertex of tFNA. MiR‐143‐3p was modified to the 5’ end of S3 to form a new single strand (Table S1, Supporting Information). The synthesis process is based on the principle of dynamic self‐assembly of nucleic acids. To demonstrate that we successfully constructed tFNAs and tFNAs‐143, we identified the integrity of nucleic acid chain hybridization and the molecular weight of the corresponding nucleic acid chain by agarose gel electrophoresis (AGE). Respectively, Figure 1B showed the molecular weight positions of S1, S2, S3, S3‐miR‐143‐3p, S4, tFNAs, and tFNAs‐143, obviously, tFNAs or tFNAs‐143 were longer than the other four ssDNAs. And the migration speed of tFNAs or tFNAs‐143 was significantly slower than that ssDNA. The clear and highlighted band of tFNAs‐143 indicated that we granted a successful fabrication of the miRNA‐functionating tetrahedral framework nucleic acid. Afterwards, we detected the surface morphology characteristics of tFNAs and tFNAs‐143 using transmission electron microscopy (TEM) and atomic force microscopy (AFM). Consistent with previous research, as we observed from TEM imaging (Figure 1C), the shape of the tFNAs or tFNAs‐143 was approximately triangular with well‐dispersed phenomenon. Additionally, the results from AFM revealed that tFNAs or tFNAs‐143 manifested a framework morphology with dimensions of 10–20 nm (Figure 1D).

To verify whether the functionalization of miR‐143‐3p changes the physicochemical properties of the tetrahedral framework nucleic acid, dynamic light scattering (DLS) tests were conducted to explore the zeta potential of tFNAs‐143. TFNAs and tFNAs‐143 were negatively charged, and the zeta potential was approximately −2.25 mV and −12.7 mV, respectively. Altogether, the results verified that the tFNAs‐143 were successfully assembled. The basis for the biological function of tFNAs‐143 is that they can efficiently achieve transmembrane action and enter the cell. We employed immunofluorescence staining to track the cellular uptake of tFNAs‐143. First, we labeled the S1‐ssDNAs with Cy5 (Cy5‐S1), and then synthesized Cy5‐labeled Cy5‐tFNAs and Cy5‐labeled Cy5‐tFNAs‐143 using Cy5‐S1. After incubation for 24 h, the cell skeleton was stained with phalloidin and observed by confocal laser microscopy for cellular uptake. As illustrated by the immunofluorescence staining in Figure 1F, Cy5‐tFNAs and Cy5‐tFNAs‐143 were distributed in the cytoplasm of condylar chondrocytes, and almost no Cy5 fluorescence was found outside the cell membrane, implying that Cy5‐labeled tFNAs‐143 successfully penetrated the cell membrane and entered the cell. Taken together, these findings confirmed the successful construction of tFNAs‐143, which penetrated the chondrocytes membrane to enter the cells.

### 2.2 TFNAs‐143 Have No Toxic Effects on Chondrocytes In Vitro and Show No Obvious Organ Toxicity In Vivo

**Figure 2:**
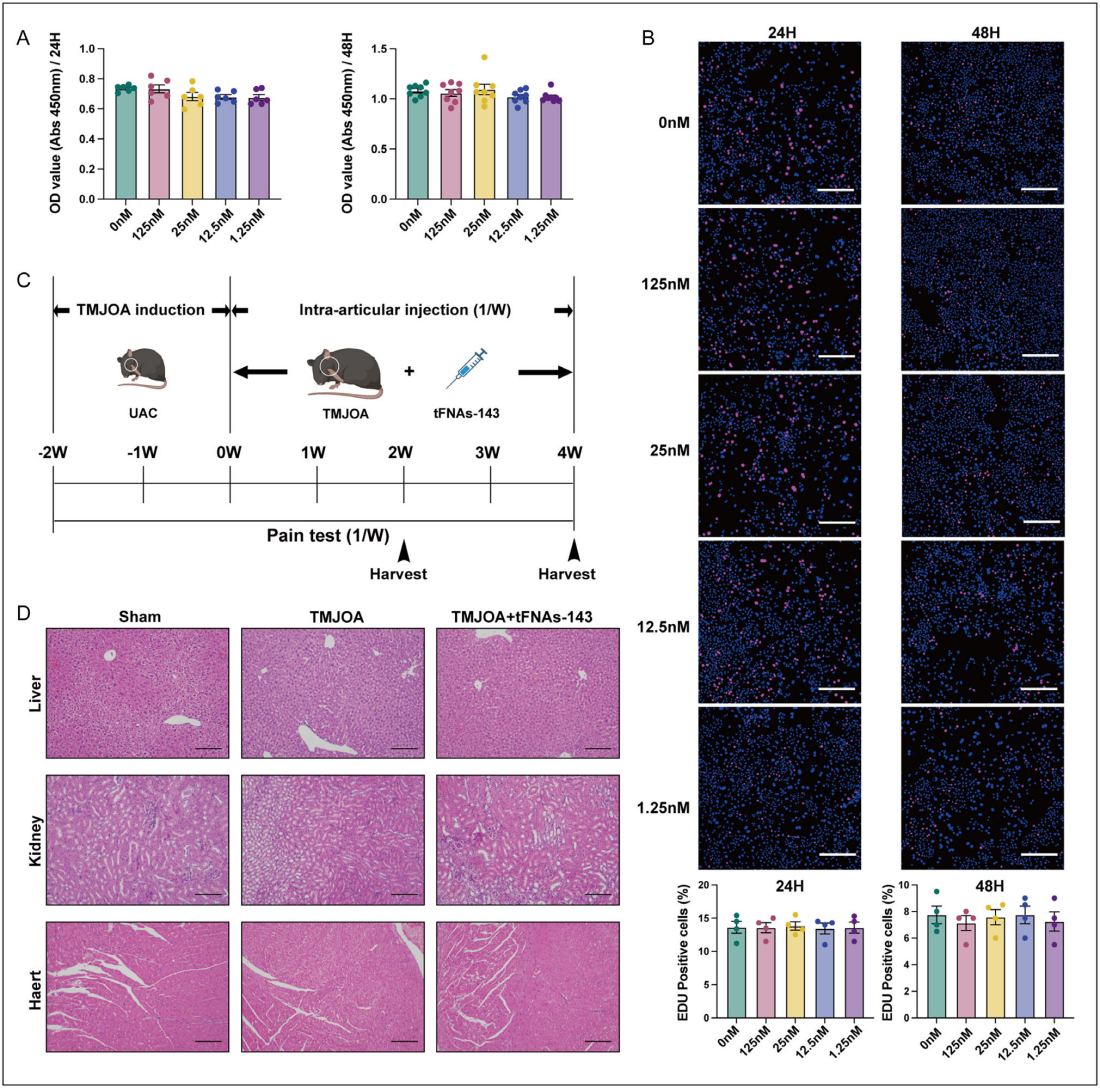
TFNAs‐143 have no toxic effects on chondrocytes in vitro and show no obvious organ toxicity in vivo. A) Viability of chondrocytes treated with tFNAs‐143 was tested by CCK‐8 assay. B) Cell proliferation of chondrocytes treated with tFNAs‐143 was tested by EdU assay. Scale bar: 100 μm. C) Schematic diagram showing the construction of TMJOA models and the schedule of tFNAs‐143 application. D) HE staining showing changes in cell morphology and tissue structure of liver, kidney, and heart after the intervention of tFNAs‐143. One‐way analysis of variance (ANOVA) for multiple comparisons between groups. Scale bar: 100 μm.

To investigate whether the tFNAs‐143 had toxic effects during application, the cytotoxicity of tFNAs‐143 was assessed through a cell counting kit‐8 (CCK‐8) assay and 5‐Ethynyl‐2'‐deoxyuridine (EdU) assay at concentrations of 125, 25, 12.5, and 1.25 nM (**Figure** **2**A,B). After 24‐ or 48‐hour treatment with tFNAs‐143, no apparent decrease in cell viability occurred, manifesting no significant difference of optical density (OD) values between the intervention group and the blank group (Figure 2A). Consistently, as seen in Figure 2B, the number of EdU^+^ cells did not show a pronounced decrease after tFNAs‐143 intervention at various concentrations. The data indicated that the tFNAs‐143 exhibited no toxic effects on the chondrocytes in vivo.

To explore the effect of the tFNAs‐143 on TMJOA, we injected the tFNAs‐143 into the TMJ cavity of the mice (Figure 2C). During application, HE staining was employed to evaluate the toxicity of tFNAs‐143 on the animals’ tissue morphology and structure of organs such as heart, kidney, and liver (Figure 2D). Hepatocytes were arranged radially around the central vein and had abundant cytoplasm and round nuclei located in the center without significant enlargement in three groups. Besides, liver lobules were clearly visible. In Figure 2D, we could see that the glomerular size and morphology of the TMJOA group and TMJOA+tFNAs‐143 group were normal, with clear boundaries. In all groups, there were numerous proximal tubules composed of cuboidal epithelial cells located at the base. In addition, the brushes inside the renal tubules remained intact without significant dilation, and there was no significant inflammatory cell infiltration in the renal interstitium of the TMJOA group and the TMJOA+ tFNAs‐143 group. As shown in Figure 2D, in the Sham group, the slender cylindrical muscle fibers were interconnected in the longitudinal section to form a network. And in the transverse section, muscle fibers appeared as short cylindrical or polygonal shapes. Between the muscle fibers, there were some capillaries and connective tissue. The same histological features were also observed in the stained sections of the hearts from the other two groups. These above data indicated that tFNAs‐143 had no significant organ toxicity effects on TMJOA mice in vivo.

### 2.3 TFNAs‐143 Alleviate TMJOA‐Induced Progressive Condylar Cartilage Destruction and Regulate ECM Activity

**Figure 3:**
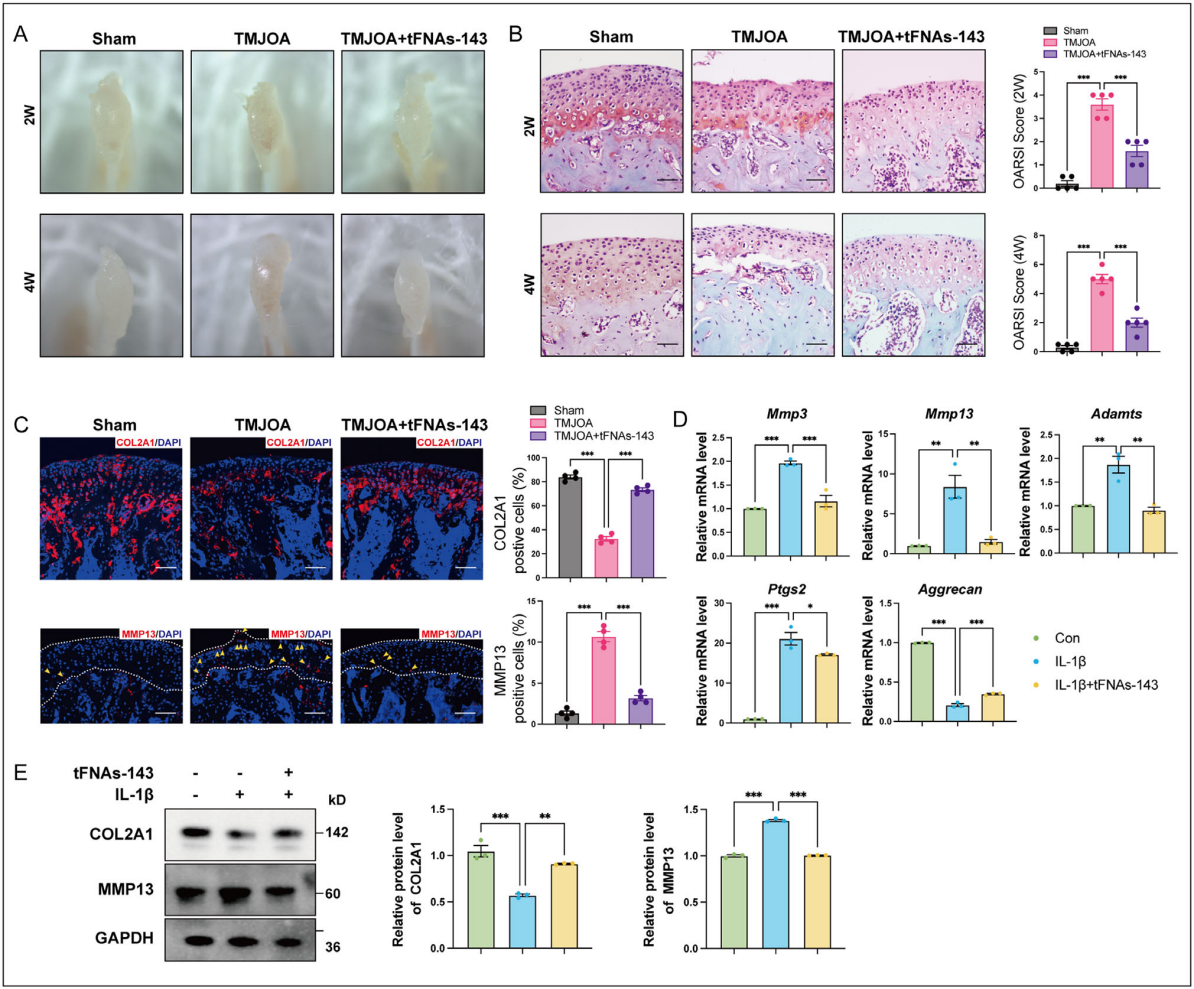
TFNAs‐143 alleviate TMJOA‐induced progressive condylar cartilage destruction and regulate ECM activity. A) Stereomicroscope displaying changes in joint disc morphology after the intervention of tFNAs‐143. B) Safranin‐O/Fast green staining, OARSI scoring system showing the degradation in condylar cartilage. Scale bar: 100 μm. C) Immunofluorescence staining and quantitative analysis for COL2A1 and MMP13 in condylar cartilage postindicated treatment. Arrow heads indicated positive cells. Scale bar: 100 μm. D) Quantitative RT‐PCR analysis of the mRNA levels of the ECM metabolism‐related genes of condylar chondrocytes after indicated treatment. E) Western blot analyses of the protein levels of the COL2A1 and MMP13 in condylar chondrocytes after indicated treatment. One‐way ANOVA for multiple comparisons between groups. Data are represented as mean ± SEM. *P < 0.05, **P < 0.01, ***P < 0.001.

Progressive degradation of condylar cartilage is one of the most prominent pathological features of TMJOA.^[^ ^37^ ^]^ To explore the role of the tFNAs‐143 in TMJOA progression, we evaluated the dynamic changes of cartilage in UAC‐induced TMJOA models. Under the stereomicroscope, the surface of the condylar cartilage in the Sham group was smooth, with uniform morphology and no obvious vascularization (**Figure** **3**A). However, whether in the 2 or 4 weeks, in the TMJOA group, the condylar cartilage became thinner and had an irregular surface. Meanwhile, due to the thinning of cartilage, the blood vessels in the subchondral bone could be seen clearly. It is worth noting that in the 2 weeks, the vascularization of the condylar cartilage in the TMJOA+ tFNAs‐143 group was reduced, although there was still some damage to the cartilage surface. After 4 weeks of tFNAs‐143 application, the thickness of the condylar cartilage increased, the surface restored smoothness. Meanwhile, the phenomenon of vascularization disappeared. Safranin‐O/Fast green staining is a classic morphological evaluation method for articular cartilage.^[^ ^38^ ^]^ It showed that the normal surface of the condylar cartilage was intact and continuous in the Sham group (Figure 3B). After the induction of TMJOA, the cartilage apparently thinned and even showed small fissures. And the chondrocytes in the proliferative layer of cartilage were disordered and arranged in clusters. At week 4, the destruction of cartilage gradually increased in severity with even local cartilage exfoliation. Furthermore, the score of osteoarthritis research society international (OARSI) dramatically gone up in the TMJOA group after TMJOA induction for 2 or 4 weeks. However, articular cartilage degradation was attenuated after the tFNAs‐143 application at 2‐ and 4‐weeks, together with a significantly decreased OARSI score.

The metabolism of ECM in chondrocytes is closely related to cartilage damage in the progression of osteoarthritis.^[^ ^39^ ^]^ Collagen type II Alpha 1 (COL2A1) is an ECM anabolism‐related molecule, while MMP13 is an ECM degradation metabolism‐related molecule. Notably, immunofluorescence staining revealed that, compared with the Sham group, the number of COL2A1 positive cells of the condylar cartilage sharply dropped in the TMJOA group (Figure 3C). But the tFNAs‐143 reversed the decrease of COL2A1 positive cells in TMJOA. Conversely, there was a significant increase in the MMP13 positive cells of the cartilage in the TMJOA group (Figure 3C). After the injection of tFNAs‐143 into TMJ joint cavity, the number of MMP13 positive cells in the condylar cartilage of TMJOA mice remarkably reduced. Furthermore, we examined the expression of ECM metabolism‐related genes after the tFNAs‐143 application. Consistent with in vivo data, the mRNA levels of catabolic genes (*Mmp3*, *Mmp13*, *Adamts*, and *Ptgs2*) significantly increased and the mRNA level of synthetic metabolic gene (*Aggrecan*) significantly decreased (Figure 3D). Notably, after applying the tFNAs‐143, the above trend was reversed. Besides, at the protein expression level, compared to the IL‐1β group, the expression of COL2A1 markedly upregulated, but the expression of MMP13 was downregulated in the IL‐1β+ tFNAs‐143 group (Figure 3 E). Accordingly, the tFNAs‐143 had been shown to significantly promote ECM synthesis metabolism and inhibit ECM catabolism.

In summary, the tFNAs‐143 alleviated progressive condylar cartilage destruction in TMJOA and regulated ECM activity via the promotion of ECM synthetic metabolism and the inhibition of ECM catabolic metabolism.

### 2.4 TFNAs‐143 Hinder the TMJOA‐Driven Abnormal Remodeling of Subchondral Bone

**Figure 4:**
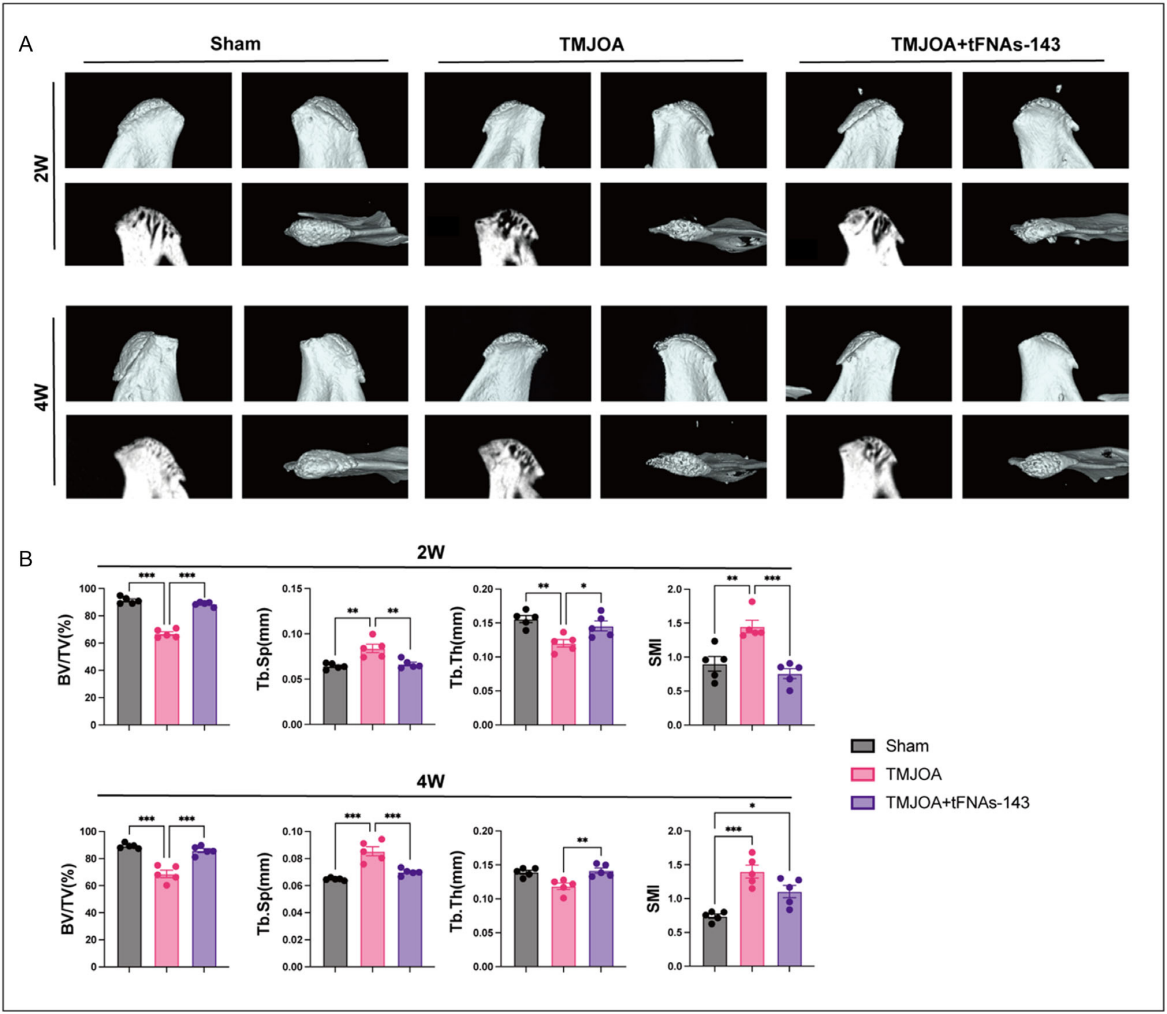
TFNAs‐143 hinder the TMJOA‐driven abnormal remodeling of subchondral bone. A) 3D reconstruction of the condyles. B) The ratio of BV/TV, trabecular space (Tb. Sp), trabecular thickness (Tb. Th), and SMI in subchondral bone. One‐way ANOVA for multiple comparisons between groups. Data are represented as mean ± SEM. *P < 0.05, **P < 0.01, ***P < 0.001.

Uncoupled remodeling of subchondral bone is another typical feature that contributed to TMJOA.^[^ ^40^, ^41^ ^]^ To elucidate the effect of the tFNAs‐143 on subchondral bone remodeling in TMJOA, we reconstructed the three‐dimensional morphology of the condyle using micro‐computed tomography (Micro‐CT) and analyzed the subchondral bone density of the condyle. From 3D reconstruction, the surface of the condyle became uneven, osteoporosis appeared, and the shape of the condyle also changed in the TMJOA group. However, the condyle morphology was improved after tFNAs‐143 administration, showing a smooth line and dense bone in 2 or 4 weeks (**Figure** **4**A). Furthermore, as shown in Figure 4B, there was a marked reduction of the ratio of bone volume to tissue volume (BV/TV) in the TMJOA group compared with the Sham group at 2‐ and 4‐weeks. Nevertheless, BV/TV significantly increased after tFNAs‐143 treatment (Figure 4B). Trabecular thickness (Tb.Th) showed a consistent trend among the groups in the 2 weeks. Of note, at 2 or 4 weeks, the trabecular separation (Tb.Sp) showed a rise after TMJOA induction. But the Tb.Sp significantly elevated in TMJOA+tFNAs‐143 group at both time points compared with TMJOA group. Moreover, compared to the Sham group, structure model index (SMI) increased prominently in the TMJOA group at week 2 or 4, which indicated that osteoporosis occurred in the subchondral bone and the bone trabeculae transformed from plate‐like to rod‐shaped. It was found that the SMI of the TMJOA+tFNAs‐143 group observably reduced compared with TMJOA group only at 2‐week. Overall, these data suggested that the tFNAs‐143 significantly inhibited abnormal subchondral bone remodeling in TMJOA.

### 2.5 TFNAs‐143 Relieve the Pain of the TMJ Region in TMJOA Mice

**Figure 5:**
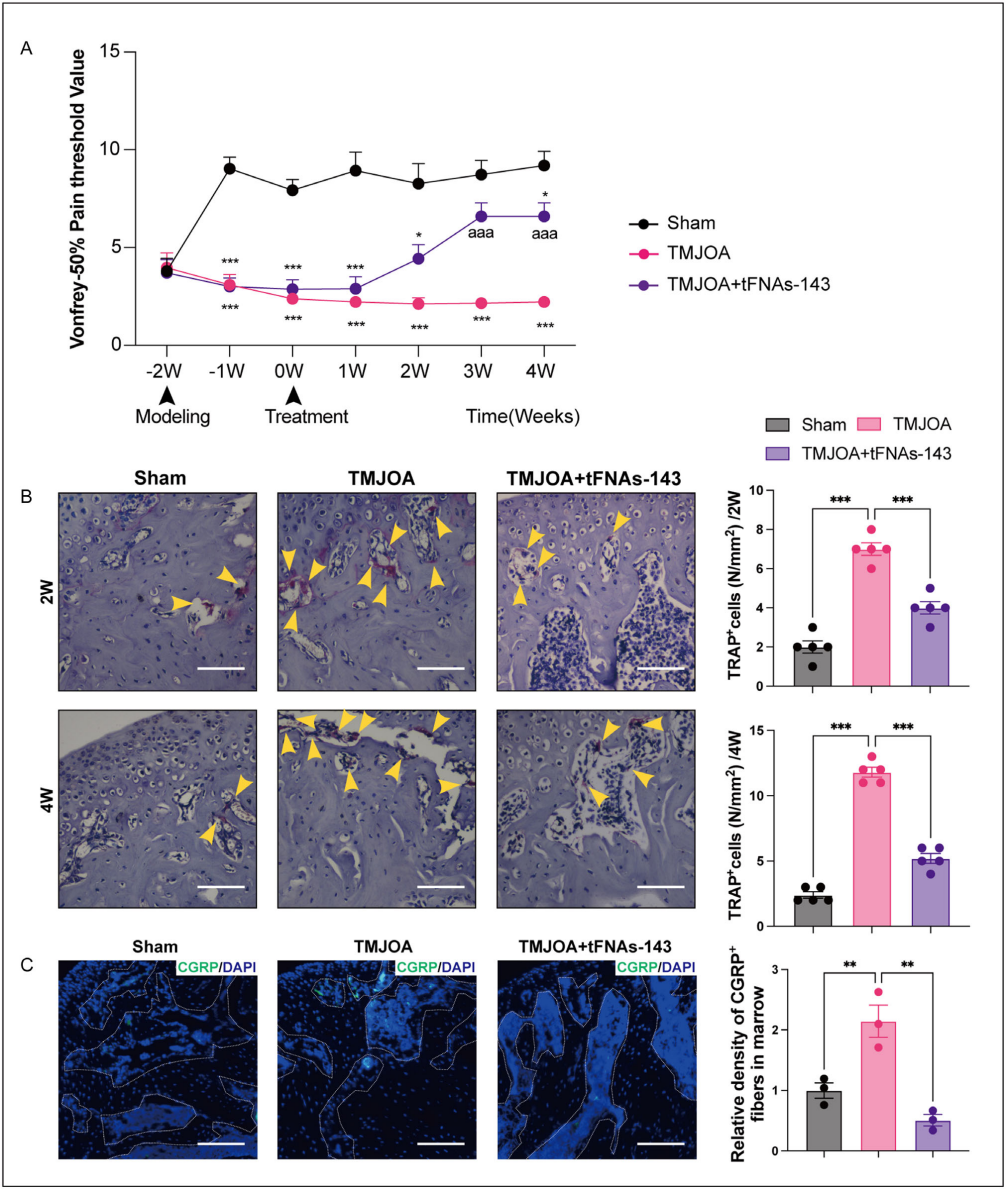
TFNAs‐143 relieve the pain of the TMJ region in TMJOA mice. A) Measurement of the pain threshold value in TMJ region by Von Frey monofilaments testing. Compared to Sham group, * *P* < 0.05, *** *P* < 0.001; compared to TMJOA group, aaa *P* < 0.001. B) TRAP staining in subchondral bone. Scale bar, 100 μm. Arrowheads indicate positive cells. C) Immunofluorescence staining for CGRP in subchondral bone. Scale bar, 100 μm. One‐way ANOVA for multiple comparisons between groups. Data are represented as mean ± SEM. **P < 0.01, ***P < 0.001.

To explore whether the tFNAs‐143 participate in the pain management in TMJOA, we, first, evaluated the pain threshold of TMJ region through Von Frey monofilaments testing. As displayed in **Figure** **5**A, before modeling, there was no significant difference in base values of pain threshold between the three groups. After TMJOA induction, compared with the Sham group, the pain threshold of the TMJOA group dramatically dropped (Figure 5A). After the application of the tFNAs‐143, the pain threshold of the TMJOA+tFNAs‐143 group raised at 2 weeks, which were higher than that in the TMJOA group. It was not until week 3 that the pain threshold of the TMJOA+tFNAs‐143 group significantly rose compared with the TMJOA group but remained lower than that of the Sham group.

Osteoclast‐induced subchondral remodeling plays a crucial role in the development of pain hypersensitivity and the emergence and innervation of sensory nerves in OA progression.^[^ ^42^ ^]^ Tartrate resistant acid phosphatase (TRAP) staining is commonly used to evaluate osteoclast activity. In Figure 5B, compared to the Sham group, TRAP staining showed that the number of osteoclasts lining the trabecular bone strikingly increased in the TMJOA group. Concurrently, TMJOA+tFNAs‐143 group displayed a significant decline in the number of osteoclasts compared with the TMJOA group. This also revealed, at 2 or 4 weeks, the tFNAs‐143 significantly down‐regulated the activity of osteoclasts in subchondral bone.

To further verify whether the tFNAs‐143 could alleviate the pain of TMJ region induced by TMJOA, we subsequently investigated the expression of CGRP (a potent vasodilator that causes pain sensibilization) in condylar subchondral bone.^[^ ^42^ ^]^ Immunofluorescence staining showed abnormal distribution of CGRP near the surface of bone trabeculae in the TMJOA group, with an outstanding enhancement in the number and density of nerve endings (Figure 5C). However, the nerve endings on the trabecular surface of the subchondral bone in the TMJOA+tFNAs‐143 group were less dense than those in the TMJOA group. Collectively, these findings implied that the tFNAs‐143 alleviated the pain of TMJOA by increasing pain threshold, down‐regulating CGRP expression in bone trabeculae, and osteoclast activity in subchondral bone.

### 2.6 A Close Correlation between the Functions of ECM Metabolism, Plasma Membrane Transformation, and Lipid Metabolism and the Alleviation of TMJOA by the tFNAs‐143

**Figure 6:**
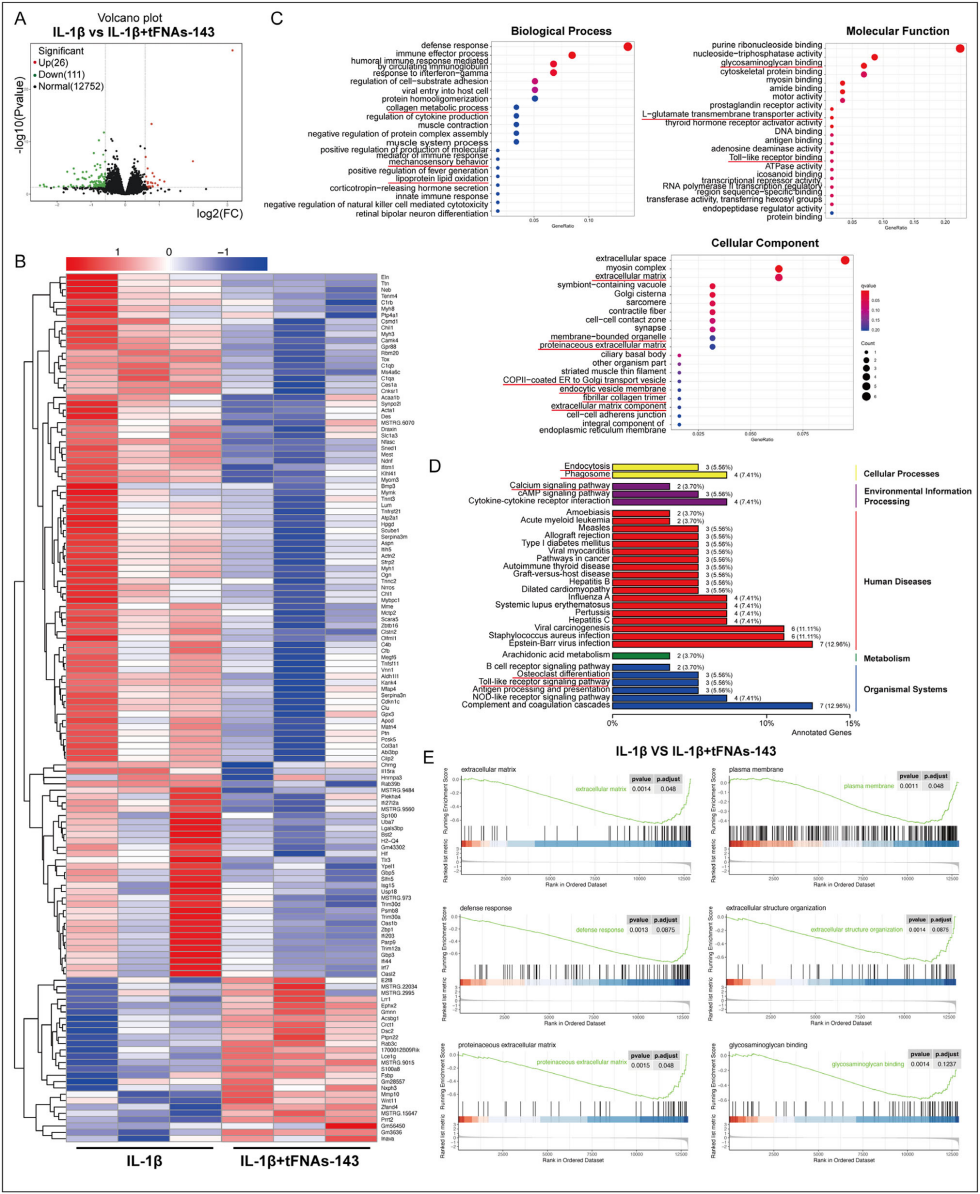
A close correlation between the functions of ECM metabolism, plasma membrane transformation, and lipid metabolism and the alleviation of TMJOA by the tFNAs‐143. A) Volcano plot of the DEGs in condylar chondrocytes after indicated treatment. B) Heatmap analysis of DEGs in condylar chondrocytes after indicated treatment. C) GO enrichment analyses of the DEGs. D) KEGG pathway analyses of the DEGs. E) GSEA analyses of the DEG.

To further elucidate the potential mechanism by which the tFNAs‐143 ameliorated TMJOA, we utilized high‐throughput transcriptome sequencing (RNA‐sequencing) to study gene function and structure at the global level, obtain differentially expressed genes (DEGs), and reveal possible biological processes. As shown in **Figure** **6**A, after the tFNAs‐143 intervention on cartilage stimulated by IL‐1β, 137 differentially expressed genes were identified in chondrocytes, including 26 upregulated DEGs and 111 downregulated DEGs. The heatmap displayed some DEGs between the IL‐1β group and the IL‐1β+tFNAs‐143 group (Figure 6B).

Then, the biological processes (BP), cellular components (CC), molecular functions (MF), and signaling pathways involved in the regulation of cartilage by the tFNAs‐143 were determined by Gene Ontology (GO) and Kyoto Encyclopedia of Genes and Genomes (KEGG) analyses. GO analysis showed that the identified DEGs are associated with “Collagen metabolic process”, “Mechanosensory behavior”, and “Lipoprotein lipid oxidation” in BP module, “Glycosaminoglycan binding”, “L‐glutamate transmembrane transporter activity”, and “Toll‐like receptor binding” in MF module, and “Extracellular matrix”, “Membrane‐bounded organelle”, “Proteinaceous extracellular matrix”, “COPII‐coated ER to Golgi transport vesicle”, “Fibrillar collagen trimer”, “Endocytic vesicle membrane”, and “Extracellular matrix component” in CC module (Figure 6C). Besides, in the KEGG analysis, the DEGs were also found to be involved in the “Endocytosis”, “Phagosome”, “Calcium signaling pathway”, “Osteoclast differentiation”, and “Toll‐like receptor signaling pathway” (Figure 6D). The results of DEGs functions suggested that tFNAs‐143 improved cartilage state in inflammatory environment might be involved in chondrocytes extracellular matrix metabolism, lipid metabolism, and plasma membrane transformation processes. Furthermore, gene set enrichment analysis (GSEA) is used to evaluate the distribution trend of a pre‐defined gene set in gene expression data to determine its contribution to phenotype, thus better capturing the subtle but coordinated effects of changes on biological pathways. Consistently, as seen in Figure 6E, the gene set enriched by GSEA included “Extracellular matrix”, “Plasma membrane”, “Glycosaminoglycan binding”, etc. As expected, the results revealed that the tFNAs‐143 had an impact on ECM metabolism and membrane structure of condylar cartilage in TMJOA.

### 2.7 TFNAs‐143 Reverse IL‐1β‐Enhanced Ferritinophagy in Condylar Chondrocytes

**Figure 7:**
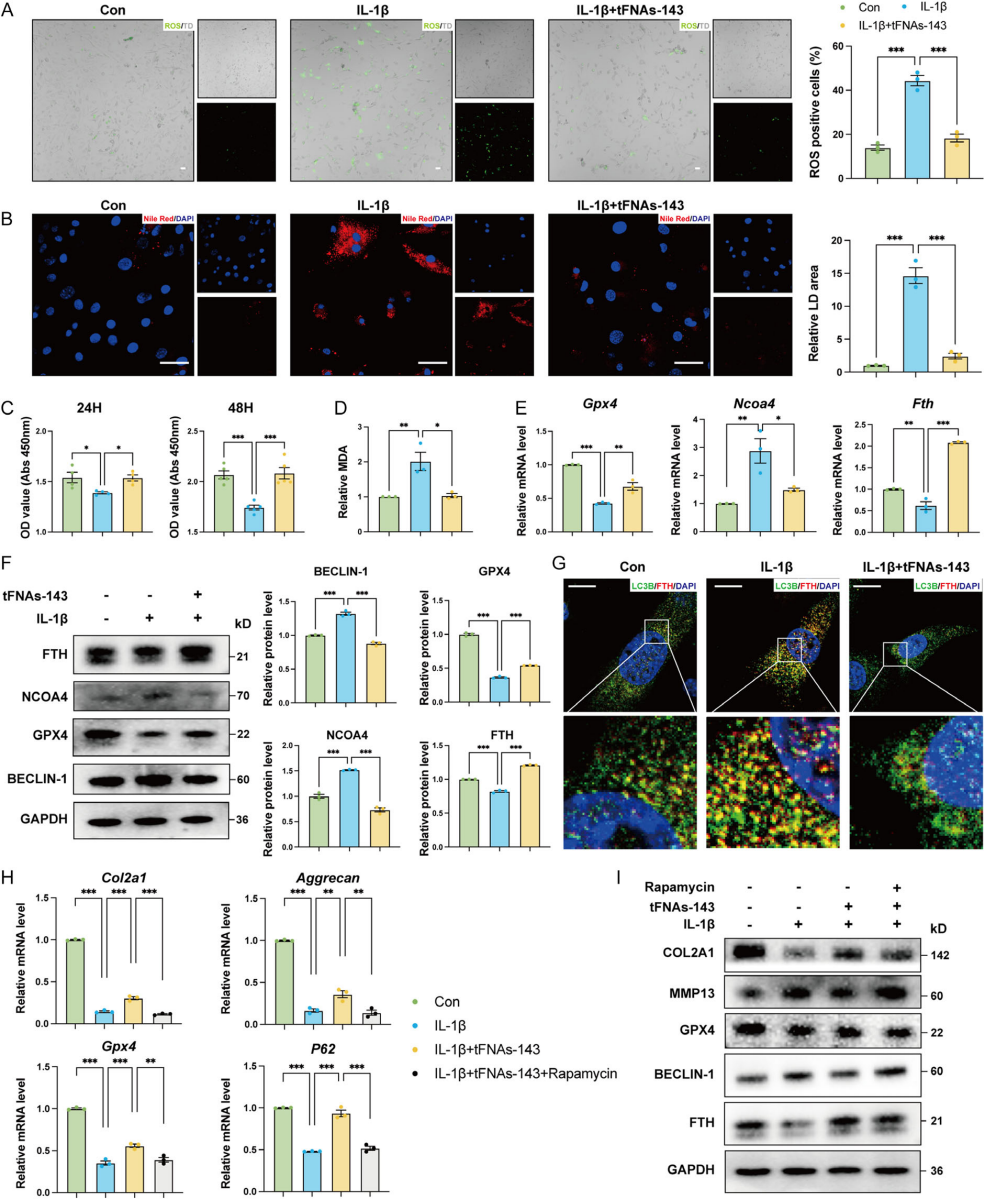
TFNAs‐143 reverse IL‐1β‐enhanced ferritinophagy in condylar chondrocytes. A) ROS staining and analyses in condylar chondrocytes after indicated treatment. Scale bar: 100 μm. B) Nile Red staining and analyses in condylar chondrocytes after indicated treatment. Scale bar: 100 μm. C) Viability of condylar chondrocytes was tested by CCK‐8 assay after indicated treatment. D) Relative MDA measurement in condylar chondrocytes 48 h after indicated treatment. E) Quantitative RT‐PCR analysis of the mRNA levels of the ferritinophagy‐related genes of condylar chondrocytes after indicated treatment. F) Western blot analyses of the protein levels of the ferritinophagy‐related genes of condylar chondrocytes after indicated treatment. G) Immunofluorescence staining for LC3B and FTH of condylar chondrocytes after indicated treatment. H) Quantitative RT‐PCR analysis of the mRNA levels of the ferritinophagy‐related genes and the ECM metabolism‐related genes of condylar chondrocytes after indicated treatment. I) Western blot analyses of the protein levels of the ferritinophagy‐related genes the ECM metabolism‐related genes of condylar chondrocytes after indicated treatment. One‐way ANOVA for multiple comparisons between groups. Data are represented as mean ± SEM. *P < 0.05, **P < 0.01, ***P < 0.001.

Ferroptosis is a novel form of programmed cell death that is iron dependent and driven by lipid peroxidation.^[^ ^32^ ^]^ Numerous studies have shown that during osteoarthritis, ferroptosis regulates the ECM metabolism of chondrocytes.^[^ ^29^, ^43^, ^44^ ^]^ To further clarify whether the tFNAs‐143 regulate ECM metabolism by reducing ferroptosis sensitivity, we performed immunofluorescence stanning to detect and quantify the changes of reactive oxygen level (ROS) under the inflammatory microenvironment of chondrocytes after intervention (**Figure** **7**A). Reactive oxygen species (ROS) positive chondrocytes drastically increased after IL‐1β application. Nevertheless, tFNAs‐143 sharply reduced the ROS level. Further, there were more red lipid droplet accumulation in the IL‐1β group compared to that in the control (CON) (Figure 7B). Nucleic acid tadpoles significantly inhibited the generation of lipid droplets in chondrocytes. Subsequently, we used CCK‐8 experiment to detect cell viability. Whether at 24 or 48 h, the activity of chondrocytes prominently decreased in an inflammatory environment, while nucleic acid tadpoles could increase cell activity (Figure 7C). In addition, compared with the IL‐1β group, the IL‐1β+tFNAs‐143 group had lower malonaldehyde (MDA) relative level (Figure 7D). Ferroptosis is also manifested by a decrease in the core enzyme GPX4, which regulates the antioxidant system.^[^ ^45^ ^]^ We validated the enhancement of chondrocytes GPX4 expression by tFNAs‐143 at the molecular level using quantitative real‐time polymerase chain reaction (qRT‐PCR) and western blot. As shown in Figure 7E, after tFNAs‐143 was introduced into chondrocytes induced by IL‐1β, the mRNA and protein level of GPX4 elevated sharply. The above results suggested that the tFNAs‐143 significantly reduced the level of lipid peroxidation in chondrocytes under inflammatory conditions and was correlated with possible ferroptosis limitation.

Previous studies have shown that ferritinophagy is the autophagic degradation of ferritin (FTH), which promotes the accumulation of cellular iron to induce ferroptosis.^[^ ^46^ ^]^ NCOA4 is a transport receptor for FTH degradation.^[^ ^47^ ^]^ To clarify whether nucleic acid tadpoles inhibit ferroptosis by regulating ferritinophagy, we evaluated the expressions of ferritinophagy‐related genes. It is worth noting that after the treatment of IL‐1β, the mRNA level of *Ncoa4* in chondrocytes strikingly upregulated but the mRNA level of *Fth* downregulated (Figure 7E). However, the tFNAs‐143 inhibited the upregulation of *Ncoa4* mRNA expression and downregulation of *Fth* expression. Consistently, tFNAs‐143 treatment was observed to downregulate the protein level of NCOA4 and downregulate the protein level of FTH chondrocytes in the IL‐1β+tFNAs‐143 group significantly compared to that in the IL‐1β group (Figure 7F). Meanwhile, as shown in Figure 7F and Supporting Information Figure 1A, the tFNAs‐143 significantly rescued the upregulation of BECLIN‐1 protein expression and the downregulation of P62 protein expression driven by IL‐1β. Consistently, after transfection with mRFP‐GFP‐LC3 virus and treatment with IL‐1β in the chondrocytes, there was apparent increase in autophagy and autophagy flux (the significant increase in red dots and yellow dots). However, after the tFNAs‐143 intervention, the phenomenon was reversed (Supporting Information Figure 1B). Moreover, we validated the inhibition of chondrocytes ferritinophagy by the tFNAs‐143 using immunofluorescence co‐staining. Specifically, he immunofluorescence staining data showed that more ferritin entered autophagosome in chondrocytes stimulated with IL‐1β, as evidenced by increased co‐localization level of LC3B and FTH (Figure 7G). But the tFNAs‐143 led to remarkable hinder of ferritinophagy, as the co‐localization level was notably dropped following the addition of nucleic acid tadpoles. In summary, these results indicated that the tFNAs‐143 significantly blocked ferritinophagy of condylar chondrocytes. Besides, we made preliminary predictions on the target genes of tFNAs‐143 in blocking ferritinophagy of condylar chondrocytes. The Venn diagram on upregulated differentially genes (DEGs) after IL‐1β intervention (Con vs IL‐1β) and downregulated DEGs after tFNAs‐143 intervention (IL‐1β versus IL‐1β+tFNAs‐143),it showed 48 DEGs (Figure 2A, Supporting Information). Using the miRDB online analysis platform, miR‐143‐3p was found the predicted binding relationships between miR‐143‐3p and the DEG, Mctp2 (Figure 2B,C, Supporting Information). Therefore, we validated the target gene through qRT‐PCR. Interestingly, the expression of Mctp2 was significantly upregulated after IL‐1β intervention and downregulated after tFNAs‐143 treatment, suggesting that it may be a target gene of the tFNAs‐143 (Figure 2D, Supporting Information).

To further confirm that ferritinophagy mediated the inhibitory effect of the tFNAs‐143 on ferroptosis in TMJOA cartilage, we performed qRT‐PCR and western blotting (WB) to detect and quantify the changes of chondrocytes ferritinophagy status after rapamycin intervention, an autophagy inducer. Compared to the IL‐1β+tFNAs‐143 group,the mRNA level of ECM metabolism‐related genes, such as *Col2a1*, *Aggrecan* and ferritinophagy‐related genes, such as *Gpx4 and P62*, declined markedly, after rapamycin was introduced into chondrocytes (Figure 7H). Meanwhile, it showed a great promotion in the protein levels of MMP13 and BECLIN‐2 in the IL‐1β+tFNAs‐143+Rapamycin group compared to the 1β+tFNAs‐143 group (Figure 7I). Conversely, the protein expression of COL2A1, GPX4, and FTH of in chondrocytes remarkably went down during Rapamycin application. These data further demonstrated that the tFNAs‐143 ameliorated TMJOA by impeding ferritinophagy‐mediated ferroptosis in condylar cartilage.

## 3 Discussion

**Figure 8:**
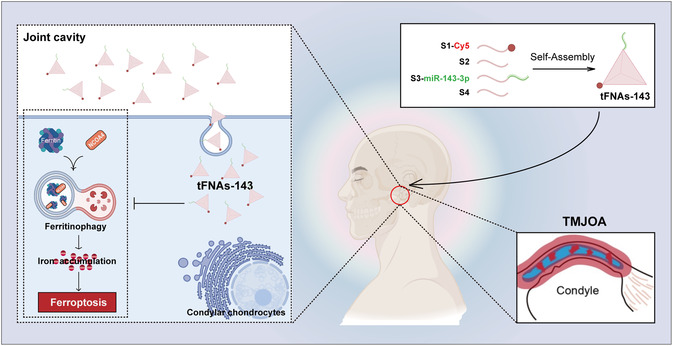
Schematic illustration of a potential nucleic acid tadpole, utilizing miR‐143‐3p functionalized tetrahedral framework nucleic acids (tFNAs‐143), to alleviate TMJOA via curbing the ferritinophagy.

The therapeutic advantages of miRNAs in temporomandibular joint osteoarthritis have received widespread attention, but its bottleneck also greatly limits its translational medicine and clinical application. By using an *in vivo* unilateral anterior occlusion (UAC) induced TMJOA model and *in vitro* IL‐1β induced microenvironment, our study uncovers the pivotal roles and the mechanisms of the rigid flexible coupling nucleic acid tadpoles functionalized by miR‐143‐3p (tFNAs‐143) in alleviating TMJOA and targeting blockade of cartilage ferritinophagy during the regulation of TMJ microenvironment (**Figure** **8**).

Nanomaterials, as excellent gene carriers and tissue repair materials, have recently achieved a breakthrough in the transformation from two‐dimensional (2D) to three‐dimensional (3D).^[^ ^48^, ^49^ ^]^ tFNAs are a type of rigid 3D nanostructure composed of DNA strands with good biocompatibility.^[^ ^50^ ^]^ They easily enter cells through endocytosis and resist various specific or nonspecific nucleases.^[^ ^51^, ^52^ ^]^ Previous studies have shown that tFNAs are a compellent nucleic acid vector for gene delivery.^[^ ^50^, ^53^ ^]^ Hyukjin Lee reported that siRNA‐modified DNA tetrahedral nanoparticles have shown promise in in vivo delivery, with a longer circulation time and tumor‐specific accumulation and can further successfully silence target genes in tumors.^[^ ^54^ ^]^ A PTK7‐targeting aptamer‐guided DNA tetrahedral nanostructure (s‐TDN) as a drug delivery system displayed enhanced cytotoxicity against PTK7‐positive CCRF‐CEM cells in acute lymphoblastic leukemia.^[^ ^53^ ^]^ MicroRNAs (miRNAs) with a length of 22 nucleotides have been reported as gene regulatory factors that act by inhibiting the translation or degradation of target mRNA.^[^ ^11^ ^]^ In the research and application of miRNAs, they face significant challenges like enzyme degradation.^[^ ^12^ ^]^ Researchers designed agonists and antagonists based on the structure of miRNAs, but these chemical preparations face the challenge of being easily recognized and degraded by the body.^[^ ^17^, ^55^ ^]^ Therefore, multiple injections are required in application to maintain the effective concentration of the formulation.

Considering the advantages of tFNAs loading, we constructed a tadpole‐shaped miR‐143‐3p functionalized framework nucleic acid system (tFNAs‐143) based on base complementary pairing and single‐strand annealing. AGE, TEM, and AFM detection further confirmed its successful synthesis (Figure 1B–D). The rigid tetrahedral framework structure of tFNAs‐143 enhanced resistance to nucleases and restricted enzyme cleavage sites with a closed structure. One of the rarest merits of the tFNAs structure is their potent endocytosis.^[^ ^50^, ^56^ ^]^ The composition infrastructure of the lipid membrane enables the cell membrane to carry a positive charge externally, which provides the possibility for endocytosis of negatively charged tFNAs.^[^ ^57^ ^]^ It has been observed to show outstanding cellular membrane affinity.^[^ ^58^ ^]^ We conducted a potential assessment on the tFNAs‐143 and found that they carry a negative potential (Figure 1E). When tracing fluorescently labeled tFNAs‐143 simultaneously, condylar chondrocytes could uptake tFNAs and tFNAs‐143 (Figure 1F). The modification of miR‐143‐3p functionalization did not affect the internalization of tFNAs by cells. We speculate that owing to the charge anisotropy attraction, negatively charged the tFNAs‐143 are allowed to approach positively charged cell membranes, boosting their internalization into cells. Meanwhile, the rigid flexible coupling mechanical properties of tFNAs (rigid) and extended chain of miRNA‐143‐3p (flexible) may also induce membrane fluctuation, promoting the internalization of tFNAs‐143 into the chondrocytes. Recently, extensive studies have documented organizational permeability is also an important consideration in the easy uptake of tFNAs.^[^ ^59^ ^]^ However, whether tFNAs‐143 can promote their internalization by affecting tissue permeability remains to be further explored in the future. A comprehensive understanding of the biological distribution, toxicity, and safety issues of materials is crucial for their transformation and application. The tFNAs‐143 have no toxic effects on chondrocytes in vitro (Figure 2A). In situ injection of tFNAs‐143 into the TMJ cavity was performed to treat TMJOA mice, there is no obvious organ toxicity in vivo (Figure 2B). This is consistent with previous research, where siRNA‐loaded tFNA was injected into the right ventricle of mice and retained in the brain for at least an hour without observed side effects in other vital organs.^[^ ^26^ ^]^ The above results indicate the superior biocompatibility of the tFNAs‐143, which lays a theoretical foundation for their application.

The study of the etiology and mechanism of TMJOA has attracted the interest of many scholars, and most researchers believe that it is caused by the combined action of systemic and local risk factors, leading to joint dysfunction and structural damage.^[^ ^60^ ^]^ Due to the incomplete elucidation of the etiology of TMJOA, clinical therapy for TMJOA mainly focuses on symptomatic treatment, such as nonpharmacological treatment, medication, and surgery.^[^ ^8^, ^61^ ^]^ Although the existing clinical treatment for TMJOA can alleviate the symptoms of OA, they cannot effectively hinder the occurrence and development of TMJOA or reverse the damage caused by the TMJOA to the joints. In the progression of TMJOA, pathological manifestations include progressive destruction of condylar cartilage, abnormal bone density driven by subchondral bone remodeling, and debilitating pain in the TMJ area.^[^ ^1^, ^2^ ^]^ We evaluated the therapeutic effect of the tFNAs‐143 on TMJOA from these three aspects. It is noteworthy that the tFNAs‐143 alleviate TMJOA‐induced cartilage damage (Figure 3). Moreover, tFNAs‐143 hinder the abnormal remodeling of subchondral bone, manifested as reversing the bone density downregulation in TMJOA mice (Figure 4). More interestingly, the tFNAs‐143 significantly boosted the pain threshold of the TMJ region and relieve the TMJ pain (Figure 5). Based on multidimensional evaluations of histology, imaging, and ethology, the results reveal highly promising therapeutic reagent of nucleic acid tadpoles in TMJOA.

More and more studies have confirmed that external trauma, excessive mechanical load on joint areas, occlusal instability, chondrocyte apoptosis, abnormal bone remodeling, neovascularization, and age‐related host adaptation decline can all cause functional structural disorders of TMJ, ultimately leading to TMJOA.^[^ ^4^, ^62^, ^63^ ^]^ The study of the etiology of TMJOA is also one of the important issues in tackling this disease at present. Under the influence of external abnormal factors, chondrocytes continuously secrete cytokines and various proteases, leading to the degradation of ECM in chondrocytes.^[^ ^40^, ^60^ ^]^ The dynamic balance of synthesis and catabolism of ECM is vital for maintaining the integrity and normal function of joints. It is worth noting that RNA‐sequencing in this study reveals the close correlation between the functions of ECM metabolism and the alleviation of TMJOA by the tFNAs‐143 (Figure 6). This indicates that miR‐143‐3p functioning tetrahedral framework nucleic acid may be a vital ECM regulator. The classic inhibitor of ferroptosis, Erastin, reversed the cytotoxicity and lipid accumulation caused by IL‐1β in vitro, and rescued cartilage lesions caused by OA.^[^ ^64^ ^]^ D‐mannose inhibits the ferroptosis of chondrocytes through HIF‐2α‐dependent pathways, thereby alleviating the degradation and damage of ECM.^[^ ^29^ ^]^ Interestingly, RNA‐sequencing from our study suggests that the identified DEGs are associated with “Lipoprotein lipid oxidation” in BP module, “Membrane‐bounded organelle”,“COPII‐coated ER to Golgi transport vesicle”, and “Endocytic vesicle membrane” in CC module, and “Endocytosis” and “Phagosome” in the KEGG analysis (Figure 6). There is the close relationship between the functions of plasma membrane transformation or lipid metabolism and the relieving TMJOA by the tFNAs‐143 (Figure 6). Furthermore, the results of in vitro studies suggested that tFNAs‐143 significantly inhibited sensitivity to ferroptosis in chondrocytes under the inflammatory microenvironment. The main characteristic of ferroptosis is iron dependence and lipid peroxidation reaction.^[^ ^30^ ^]^ Ferritinophagy is one of the key mechanisms for maintaining intracellular iron ion homeostasis.^[^ ^47^ ^]^ This indicates that it is an upstream mechanism for inducing ferroptosis. Previous studies demonstrated that NCOA4 could interact with ferritin and increase autophagic degradation of ferritin and iron levels, which caused chondrocyte ferroptosis and ECM degradation.^[^ ^65^ ^]^ Interestingly, immunofluorescence co‐staining experiments showed that nucleic acid tadpoles remarkably downregulated the co‐localization of ferritin and autophagosome marker proteins (Figure 7G), suggesting the inhibition of ferritinophagy. Moreover, Rapamycin, an autophagy inducer, eliminated the inhibitory effect of tFNAs‐143 on ferritinophagy in chondrocytes. Our data further demonstrated that the tFNAs‐143 ameliorated TMJOA by impeding ferritinophagy‐mediated ferroptosis in condylar cartilage.

There are several recognized limitations to our study. First, in this study, we have demonstrated that the tFNAs‐143 can be internalized by chondrocytes to exert TMJOA therapeutic effects by impeding ferritinophagy‐mediated ferroptosis. However, we have not yet fully revealed the targets and specific mechanism pathways by which the tFNAs‐143 regulate ferritinophagy process. This will be the subject of future investigations. Second, there is a great unfulfilled medical need for a miRNA‐modifying biological reagent to combat TMJOA. Although we have demonstrated the feasibility of using the tFNAs‐143 to treat TMJOA, this is only the first step in translating our research into clinical applications. Future experiments should use large animal models to test the efficacy of the tFNAs‐143 in TMJOA. Further investigation on appropriate dosages and administration methods of the tFNAs‐143 will promote the clinical application. Thirdly, although miRNA therapy has broad prospects, there are issues such as off‐target effects and immune responses. Off‐target effects are one of the main challenges, as miR‐143‐3p can target multiple mRNA transcripts, and delivered miRNAs may affect multiple cellular pathways and interfere with normal cellular function. Besides, immune response is also a key issue, miRNA therapy may unexpectedly activate local and systemic immune responses. Therefore, in the future, we should further explore strategies for precise targeting and immune compatibility of the tFNAs‐143.

In conclusion, we have successfully synthesized a promising nanoscale nucleic acid system, tFNAs‐143, which can alleviate temporomandibular joint osteoarthritis (Figure 8). They prepared that the tFNAs‐143 have the characteristics of rigid flexible coupling and structural stability, which can be internalized by chondrocytes, and exhibit excellent biocompatibility. MiR‐143‐3p functionalized tetrahedral framework nucleic acids mitigate TMJOA by inhibiting cartilage ferritinophagy and reducing susceptibility to ferroptosis. Our study will innovate the strategy of miRNAs delivery therapy for TMJOA, deepen the theoretical understanding of the pathogenesis of TMJOA, and provide a solid theoretical basis for the development and application of new therapeutic strategies for TMJOA.

## 4 Experimental Section

4.1

4.1.1

##### Synthesis and Characterization of the tFNAs‐143

As the described in previous studies,^[^ ^24^, ^25^ ^]^ the tFNAs were synthesized. The four well‐defined DNA nucleic acids single strands (ssDNA strands) (S1, S2, S3‐miR143‐3p, S4) (1 OD DNA) were synthesized by Sangon Biotech, were centrifuged, deposited, and then dissolved (final concentration, 100 μM). The four ssDNA strands were mixed with equimolar volume into TM buffer (pH = 8.0, 10 mmol L^−1^ Tris‐HCl, 50 mmol L^−1^MgCl_2_). The tFNAs‐143 were produced by complementary pairing (Figure 1A), which were denatured at 95 °C for 10 min and quickly lowered to 4 °C for 20 min. All ssDNA strands are listed in Table S1, Supporting Information. The successful formations of tFNAs‐143 were identified by 2% AGE. TEM (FEI Tecnai F20, negative staining technique, voltage 200KV) was applied to observe the morphological structure of tFNAs‐143. The surface morphology and surface properties of tFNAs‐143 were characterized by AFM using a Dimension ICON (Bruker, Germany). In addition, the zeta potential of tFNAs‐143 was detected through dynamic light scattering (DLS) (Nano ZS, Malvern, England).

##### Isolation and Culture of Condylar Primary Chondrocytes

Condylar cartilage was obtained from 10‐day‐old C57BL/6 mice. Briefly, cartilage tissues were washed with PBS for three times, dissected into 1mm3 pieces, and then digested with 2.5 mg ml^−1^ type II collagenase (Gibco) for 30 min and 0.5 mg ml^−1^ type II collagenase overnight at 37 °C. The primary chondrocytes were cultured in low glucose DMEM medium (Gibco) containing 1% penicillin‐streptomycin and 10% FBS in a humidified incubator with 5% CO_2_% and 95% humidity at 37 °C. To ensure the integrity of phenotype, we only chose condylar primary chondrocytes for the TMJOA in vitro chondrocyte model experiment. Primary condylar chondrocytes were incubated with recombinant IL‐1β (10 ng ml^−1^, Peprotech), tFNAs‐143 (12.5 nM), Rapamycin (25 nM, MCE).

##### Cellular Uptake of TFNAs‐143

The Cy5 fluorescein‐labeled ssDNA strands (S1‐Cy5) were centrifugated, dissolved, mixed with other three ssDNA strands into TM buffer, Cy5 fluorescein‐labeled tFNAs‐143 (Cy5‐tFNAs‐143) were synthesized by complementary pairing. Primary chondrocytes were plated in 24‐well plate and were co‐incubated with Cy5‐tFNAs and Cy5‐tFNAs‐143 for 24 h. The cells were fixed with 4% paraformaldehyde. Respectively, FITC‐labeled phalloidin and DAPI (Sigma, D9542) were applied to stain the cytoskeleton and nuclei. The fluorescence microscopy was used to detect the uptake of Cy5‐tFNAs and Cy5‐tFNAs‐143 by chondrocytes.

##### CCK‐8 Cell Viability Assay

Primary chondrocytes were seeded in 96‐well plates at 2000 cells/well, cultured with indicated medium at concentrations of 125, 25, 12.5, and 1.25 nM tFNAs‐143 for 24 h or 48 h. After the treatments, the chondrocytes were incubated in 100 μL of DMEM medium supplemented with 10 μL of CCK‐8 reagent (MCE) at 37 °C for 2 h to test the viability of chondrocytes. The absorbance was measured at 450 nm using the microplate reader (Synergy H1; BioTek).

##### EdU Assay

According to the manufacturer recommendations, the EDU working fluid was prepared. Primary chondrocytes were seeded in 24‐well plates, cultured with indicated medium at concentrations of 125, 25, 12.5, and 1.25 nM tFNAs‐143 for 24 h or 48 h. The chondrocytes were labeled with the EdU working fluid for 2 h and stained with DAPI. The images were acquired with fluorescence microscopy.

##### Animal Experiments

All animal experiments in this study were performed in accordance with institutional guidelines and approved by the ethics committee, Fujian Medical University (No. IACUC FJMU 2024‐Y‐1574). Eight weeks old male C57BL/6 mice weighing an average of 20 g were purchased from Wushi Experimental Animal Limited Company (Fuzhou, China). All the animals were maintained in specific‐pathogen‐free conditions on a 12‐/12‐h light/dark cycle, with free access to water and food. Mice were anesthetized with pentobarbital sodium (100 mg kg^−1^, injected intraperitoneally). According to the previous studies,^[^ ^66^ ^]^ UAC model was performed to induce TMJOA. Two metal tubes (Xinhua Pharmaceutical Limited Company, China) were bonded to the left maxillary and mandibular incisor. Soft food was fed for 2 days after the modeling, and the devices were checked daily. If the devices had fallen, they were promptly reloaded.

For experiments evaluating the effect of the tFNAs‐143 on TMJOA, animals were randomly allocated to three groups by the random isolation table (*n* = 6): Sham, TMJOA, TMJOA+tFNAs‐143. Animals in the Sham and TMJOA group received equal volume of PBS treatment. The tFNAs‐143 (10 μl 500 nM into each joint cavity, weekly) or equivalent PBS was injected into the TMJ articular cavity. Mice were sacrificed according to the schematic diagram after 2‐ and 4‐week interventions for analyses.

##### Von Frey Monofilaments Testing

The Von Frey monofilaments testing (Stoelting, Italy) is commonly applied to measure the pain experienced by animals. Used the tip of a hard plastic filament to stimulate the animal's testing area, observed the animal's reaction, and judged whether they had a painful response to the stimulus. The animals were kept calm for 30 min before the detection. The midpoint of the line connecting the corner of the eye and the ear was used as the testing area (“TMJ zone”). Starting from the minimal filament intensity, the test was repeated for three times at a 30 s interval. The intensity of the stimulus (g) was recorded when the animal underwent a transition from a calm state to mouth‐rubbing or grasping response. The average value was taken as the threshold of the TMJ zone.

##### Morphometric Analysis

Mice were sacrificed by anesthetic overdose. Bilateral TMJs were separated, fixed, and stored in 70% ethanol before subsequent processing. The morphology of the condyle was observed under a stereomicroscope (Nikon, SMZ18).

##### Micro‐CT

Micro‐CT scanning of the condyle of mice was performed by NEMO Micro‐CT (filter Al 0.2 mm, 70 kV, 114 μA, PINGSENG Healthcare Kunshan Inc, China) with the resolution of 7 μm to detect the change of the condylar subchondral bone. Three‐dimensional (3D) images were collected and reconstructed by Cruiser for morphological evaluation. Bone density was assessed by Avatar, including the ratio of BV/TV, trabecular space (Tb. Sp), trabecular thickness (Tb. Th), and SMI.

##### Histology and Immunofluorescence Staining

The condyles of mice were decalcified using EDTA for 1 month, dehydrated, embedded, and sectioned at 5 μm. According to the manufacturer's recommendations, the sections were stained with Safranin‐O/Fast green (Solarbio, China) and TRAP staining (Sigma‐Aldrich, USA). The morphology of the condylar cartilage was observed by a microscope, and the severity of the osteoarthritis‐like phenotype was assessed using the OARSI scoring system. TRAP^+^ cells in the condylar subchondral bone with three or more nuclei/cell were identified as odontoclast cells. The organs of mice were obtained, fixed, dehydrated, embedded, and sectioned. According to the manufacturer's recommendations, the sections were stained with hematoxylin‐eosin staining (HE).

For immunofluorescence staining, after blocking, frozen sections and primary chondrocytes were incubated at 4 °C overnight with primary antibodies against COL2A1(ABclonal, 1:200), MMP13(ABclonal, 1:200), CGRP (CST, 1:100), LC3B (CST, 1:100), and FTH (ABclonal, 1:200). The secondary antibodies included donkey anti‐mouse Alexa Fluor 488/555 and donkey anti‐rabbit Alexa Fluor 488/555 (all from Thermo Fisher Scientific). The nucleus was counterstained with DAPI, the images were captured with confocal microscope and analyzed with ImageJ software. Sections were blinded and scored by two experienced researchers, and the average scores were applied in statistical analyses.

##### ROS Staining, Nile Red Staining, and MDA

After the treatments with recombinant IL‐1β (10 ng ml^−1^), tFNAs‐143 (12.5 nM) for 24 h, chondrocytes were stained with DCFH‐DA (Beyotime, S0033) to measure ROS and assayed by fluorescence microscopy (Ts2R/FL, Nikon).

After the treatments with recombinant IL‐1β (10 ng ml^−1^), tFNAs‐143 (12.5 nM) for 24 h, chondrocytes were fixed and stained with 0.1 μg ml^−1^ Nile Red (MCE, HY‐D0718) for 30 min, and the nuclei were counterstained using DAPI. Images observed by a confocal microscope and analyzed with ImageJ software.

After the treatments with recombinant IL‐1β (10 ng ml^−1^), tFNAs‐143 (12.5 nM) for 24 h, the level of MDA of condylar chondrocytes was detected with a Lipid Peroxidation MDA Kit (Beyotime, China) according to the manufacturers’ protocols.

##### Double‐Labeled Adenovirus mRFP‐GFP‐LC3 Transfection

Chondrocytes were seeded on confocal Petri dishes and cultured for 24 h for attachment. Cells were then transfected with mRFP‐GFP‐LC3 adenovirus (Hanbio, China) according to the manufacturer's protocol.

##### qRT‐PCR

After the treatments with recombinant IL‐1β (10 ng ml^−1^), tFNAs‐143 (12.5 nM) for 24 h, total RNA was extracted with Trizol (Takara, Japan). The first‐strand cDNA was prepared using HiScript II Q RT SuperMix for qPCR (Yeasen Biotechnology, China). qRT‐PCR was performed using SYBR Premix Ex Taq II (Yeasen Biotechnology, China) in CFX96 Real‐Time System (Bio‐Rad). *Gapdh* RNA was used as a housekeeping control. The primer sequences are listed in Table S2, Supporting Information. The relative levels of genes expression were normalized to GAPDH and calculated using the 2^−ΔΔCt^.

##### Western Blotting Assay

The primary condylar chondrocytes lysates were extracted using RIPA buffer for 30 min (4 °C). (Beyotime, P0013B) containing phenylmethanesulfonyl fluoride (PMSF, Beyotime, ST505). The concentrations of the Proteins were detected by BCA protein assay kit (Beyotime, #P0012). The samples were heated at 100 °C for 5 min in sample buffer, separated on 10% SDS‐polyacrylamide gels, and transferred to PVDF membranes (Bio‐Rad). The membranes were blotted with 5% BSA and incubated with primary antibody at 4 °C overnight. The membranes were washed in TBST solution and incubated with the HRP‐conjugated secondary antibodies. The antibody‐antigen complexes were visualized with ECL reagents (Millipore, WBKLS0100). The following primary antibodies were applied, COL2A1 (ABclonal, 1:1000), MMP13 (ABclonal, 1:1000), LC3B (CST, 1:1000), and FTH (ABclonal, 1:1000), NCOA4 (Santa cruz, 1:1000), BECLIN‐1 (CST, 1:1000), P62 (CST, 1:1000), GPX4 (ABclonal, 1:1000), and GAPDH (ABclonal, 1:5000).

##### Sequencing and Data Analysis

Transcriptome sequencing was performed in triplicate using three independent sets of RNA preparations. Briefly, total RNA was extracted using TRIzol reagent, and 1 μg of total RNA was applied to construct sequencing libraries. The library was amplified by PCR to increase its concentration, resulting in a cDNA library for sequencing. Library samples were subjected to standardized analysis on an Illumina NovaSeq 6000 sequencer. Data quality control (QC) was performed, such as removing low‐quality reads and connector sequences (FASTP, v0.20.1). High‐quality reads to the reference genome or transcriptome (HISAT2, v2.0.4) were aligned. StringTie software (v2.1.2) for assembly of genes or transcripts is used. The version of the mouse genome we are using is Mus_musculus (GRCm39.107). The feature count was standardized using the DESeq2 package (v1.260). KOBAS software was used to test the statistical enrichment of differential expression genes in KEGG pathways. Gene Ontology (GO) enrichment analysis of the DEGs was implemented by the GOseq R packages based Wallenius noncentral hypergeometric distribution. The Sequencing and data analysis were conducted by Tsingke (Beijing, China). Genes with FDR < 0.05 & |log2(foldchange)| ≥1 found by DESeq2 were assigned as differentially expressed. Data generated during this study have been deposited in the Gene Expression Omnibus (GEO) with the accession code GSE303194.

##### Statistical Analysis

Data are presented as the mean ± standard error of at least three independent experiments. Significant differences were evaluated using two‐tailed Student's *t* tests for comparison between two groups, one‐way ANOVA for multiple comparisons of continuous measures between groups, when the variables were distributed normally, and Tukey's post hoc test was applied to determine the statistical significance between groups. When the variables were not distributed normally, the Kruskal‐Wallis's test was used. All statistical analyses were conducted using GraphPad Prism 8. *P* < 0.05 was considered statistically significant.

## Supporting Information

Supporting Information is available from the Wiley Online Library or from the author.

## Conflict of Interest

The authors declare no conflict of interest.

## Supporting information

Supplementary Material

## Data Availability

The data that support the findings of this study are available from the corresponding author upon reasonable request.
