# Supplementary material for: Nano MiRNA‐Functionating Tetrahedral Framework Nucleic Acid for Cartilage‐Targeted Ferritinophagy Modulation to Attenuate Temporomandibular Joint Osteoarthritis
Source: Small Sci. 2025 Dec 12;6(1):e2500267. doi: 10.1002/smsc.202500267 (PMC12798780; doi:10.1002/smsc.202500267)
Supplement: Supplementary file 1 — Supplementary Material [file SMSC-6-e2500267-s001.pdf]

Supplementary Materials for

Nano MiRNA-Functionating Tetrahedral Framework Nucleic Acid for Cartilage-targeted Ferritinophagy Modulation to Attenuate Temporomandibular Joint Osteoarthritis

Authors: Wenxiu Yuan [1, 2, a], Maotuan Huang [4, a], Linxin Chen [2], Sihang Chen [2], Hanyu Lin [3], Nengwen Huang [3], Yifeng Xing [3], Chengchaozi Wang [3], Jie Lu [3], Min Fu [3], Linyu Xu [2, *], Jiang Chen [3, *]

Supplementary figures

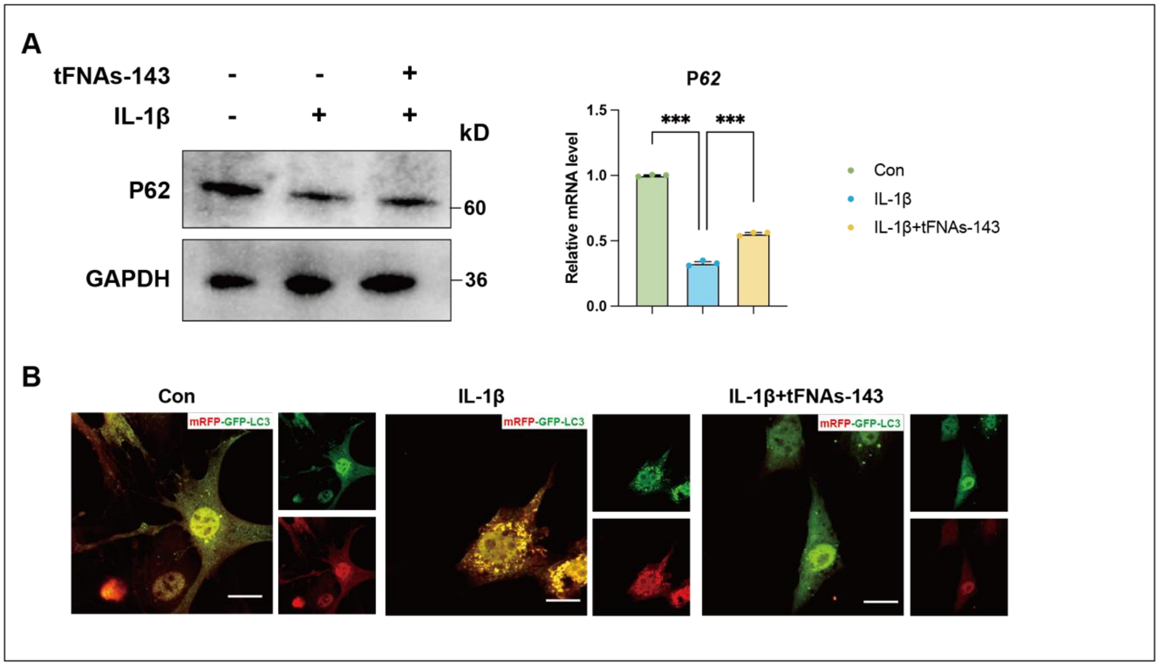

Supplementary Fig.1. TFNAs-143 reversed IL-1β-enhanced autophagy in condylar chondrocytes. (**A**) Western blot analyses of the protein levels of P62 of condylar chondrocytes after indicated treatment. (**B**) Fluorescent protein labeled LC3 monitors

autophagic flow of condylar chondrocytes after indicated treatment. One-way analysis of variance (ANOVA) for multiple comparisons between groups. Data are represented as mean ± SEM. *** $p < 0.001$.

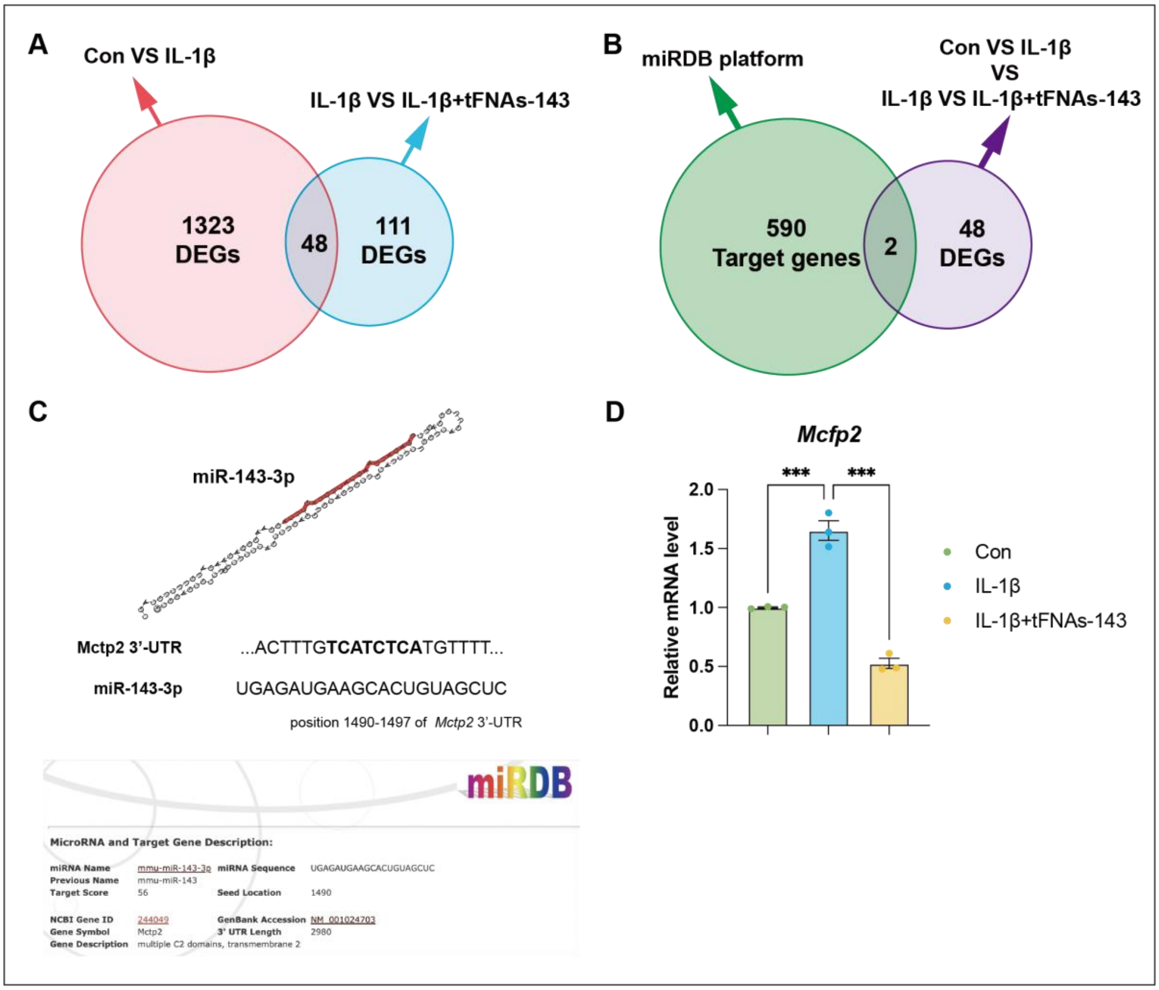

Response Fig.2. Target gene prediction of the tFNAs-143. (**A**) Venn diagram of the differentially genes (DEGs) between Con and IL-1β, and between IL-1β and IL-1β +tFNAs-143. (**B**) Venn diagram of the target genes between miRDB platform and between (Con vs IL-1β) and (IL-1β vs IL-1β+tFNAs-143). (**C**) The structure of miR-143-3p and the miR-143-3p sequence and the predicted miR-143-3p target site in the 3′ -UTR of Mctp2. (**D**) Quantitative RT-PCR analysis of the mRNA levels of the Mctp2 of condylar chondrocytes after indicated treatment. One-way analysis of variance

(ANOVA) for multiple comparisons between groups. Data are represented as mean ± SEM. *** $p < 0.001$.

Supplementary tables

Table S1. The sequences of each single-stranded DNA (ssDNA).

ssDNA	Sequences (from 5′ to 3′)
S1	ATTTATCACCCGCCATAGTAGACGTATCACCAGGCAGTTGAGACGAACATTCCTAAGTCTGAA
S2	ACATGCGAGGGTCCAATACCGACGATTACAGCTTGCTACACGATTCAGACTTAGGAATGTTCG
S3	ACTACTATGGCGGGTGATAAAACGTGTAGCAAGCTGTAATCGAGGGAAGAGCATGCCCATCC
S4	ACGGTATTGGACCCTCGCATGACTCAACTGCCTGGTGATACGAGGATGGGCATGCTCTTCCCG
S3-miR-143-3p	ACTACTATGGCGGGTGATAAAACGTGTAGCAAGCTGTAATCGAGGGAAGAGCATGCCCATCCrU//rg//rA//rg//rA//rU//rg//rA//rA//rg//rC//rA//rC//rU//rg//rU//rA//rg//rC//rU//rC//rA

Table S2. Sequences of forward and reverse primers of the housekeeper gene and related genes designed for qRT-PCR.

Gene	Primer Sequence (from 5′ to 3′)
Gapdh	F 5’- TGTTTCCTCGTCCCGTAGA -3’<br>R 5’- ATCTCCACTTTGCCACTGC -3’
Col2a1	F 5’- ACCAGATTGAGAGCATCCGC -3’<br>R 5’- CAGCCCTGGTTGGGATCAAT -3’
Mmp3	F 5’- CAATCCCTCTATGGACCTCCC -3’<br>R 5’- CCCTCCATGAAAAGACTCAGAGG -3’
Mmp13	F 5’- ACCCAGCCCTATCCCTTGAT -3’<br>R 5’- GGCCCAGAATTTTCTCCCTCT -3’
Adamts	F 5’- GCCCACCTAACGGCAAATCT -3’<br>R 5’- AGGACACCTGCGTATTTGGG -3’

Ptgs2	F 5'- TGAGTGGGGTGATGAGCAAC -3'<br>R 5'- TTCAGAGGCAATGCGGTTCT -3'
Aggercan	F 5'- GACCTGTGTGAGATCGACCA -3'<br>R 5'- GGTCGGGAAAGTGGCGATAA q -3'
Gpx4	F 5'- AATCAAGGAGTTTGCAGCCG -3'<br>R 5'- CCACGCAGCCGTTCTTATCA -3'
Ncoa4	F 5'- AGGCTATGGCTCCTGCTAGA -3'<br>R 5'- CGTCCTTAGGGCCTCCTTTG -3'
Fth	F 5'- GACCGTGATGACTGGGAGAG -3'<br>R 5'- TAGCCAGTTTGTGCAGTTCCA -3'
P62	F 5'- GGCACAGAAGACAAGAGTAACAC -3'<br>R 5'- CACCGACTCCAAGGCTATCT -3'

34
